# Evaluating dosimetric constraints for carbon ion radiotherapy in the treatment of locally advanced pancreatic cancer

**DOI:** 10.1186/s13014-020-01515-5

**Published:** 2020-05-07

**Authors:** Lien-Chun Lin, Guo-Liang Jiang, Nitin Ohri, Zheng Wang, Jiade J. Lu, Madhur Garg, Chandan Guha, Xiaodong Wu

**Affiliations:** 1 Department of Medical Physics, Shanghai Proton and Heavy Ion Center, Fudan University Cancer Hospital, Shanghai Engineering Research Center of Proton and Heavy Ion Radiation Therapy, 4365 Kangxin Road, Shanghai, 201318 China; 2 Department of Radiation Oncology, Shanghai Proton and Heavy Ion Center, Fudan University Cancer Hospital, Shanghai Engineering Research Center of Proton and Heavy Ion Radiation Therapy, Shanghai, China; 3 Department of Radiation Oncology, Albert Einstein College of Medicine and Montefiore Medical Center, 111 E 210th St, Bronx, NY 10467 USA

**Keywords:** Carbon ion, Radiotherapy, Pancreatic cancer

## Abstract

**Objective:**

To identify a safe carbon ion radiotherapy (CIRT) regimen for patients with locally advanced pancreatic cancer (LAPC).

**Methods:**

We generated treatment plans for 13 consecutive, unselected patients who were treated for LAPC with CIRT at our center using three dose and fractionation schedules: 4.6 GyRBE × 12, 4.0 GyRBE × 14, and 3.0 GyRBE × 17. We tested the ability to meet published dose constraints for the duodenum, stomach, and small bowel as a function of dose schedule and distance between the tumor and organs at risk.

**Results:**

Using 4.6 GyRBE × 12 and 4.0 GyRBE × 14, critical (high-dose) constraints could only reliably be achieved when target volumes were not immediately adjacent to organs at risk. Critical constraints could be met in all cases using 3.0 GyRBE × 17. Low-dose constraints could not uniformly be achieved using any dose schedule.

**Conclusion:**

While selected patients with LAPC may be treated safely with a CIRT regimen of 4.6 GyRBE × 12, our dosimetric analyses indicate that a more conservative schedule of 3.0 GyRBE × 17 may be required to safely treat a broader population of LAPC patients, including those with large tumors and tumors that approach gastrointestinal organs at risk. The result of this work was used to guide an ongoing clinical trial.

## Background

Pancreatic cancer is a leading cause of cancer mortality, accounting for over 300,000 deaths each year [1]. Approximately 30% of pancreatic cancer patients present with locally advanced disease, which may be defined as unresectable disease without evidence of distant metastases. Available local and systemic therapies are unlikely to convert such patients to being eligible for resection [2, 3], so treatment goals are often extension of life and preservation or improvement of quality of life. Median survival durations in clinical trials for locally advanced pancreatic cancer (LAPC) have ranged from approximately 8 to 16 months [3–9].

Primary treatment options for LAPC include chemotherapy and radiotherapy [10]. Randomized trials seeking to demonstrate the superiority of chemoradiotherapy over chemotherapy alone have yielded mixed results [3–6, 8, 9]. All of these studies utilized photon radiotherapy. Notably, in the one trial that employed an unusually high radiotherapy dose of 60 Gy, chemoradiotherapy yielded significantly shorter overall survival duration compared with chemotherapy alone [4]. This demonstrates that the therapeutic window for photon radiotherapy in this setting is likely very narrow.

Carbon ion radiotherapy (CIRT) has been studied in recent years as a strategy to improve outcomes for patients with LAPC. Compared to photon radiotherapy, CIRT confers dosimetric advantages as well as potential biological advantages [11–13]. Investigators from the National Institute of Radiological Sciences (NIRS) in Japan have recently reported encouraging results with a combination of CIRT and chemotherapy with respect to both treatment-related toxicity and treatment efficacy [14]. Of note, most patients in that study received a dose of 55.2 GyRBE in 12 fractions (4.6 GyRBE per fraction). Strict dosimetric constraints for the duodenum and stomach were applied to limit the risk of gastrointestinal toxicity, and patients with a history of obstructive jaundice requiring biliary stenting or tumor abutting the stomach or small bowel were deemed ineligible for treatment.

Our group has initiated a prospective clinical trial to establish the safety and efficacy of CIRT compared to photon radiotherapy for LAPC. Based on cases of severe GI toxicity (Grade 3 bleeding) we have observed when using fraction sizes of 4.0 GyRBE, we undertook this dosimetric study to examine our ability to achieve constraints utilized by investigators from NIRS and explore how changes in fractionation may influence our ability to generate acceptable CIRT plans.

## Methods

### Carbon ion radiotherapy planning and treatment

Patients are treated in the prone position. 4-dimensional simulation CT imaging with ten phases is obtained in the treatment position, and a gating window in the expiration phase during which target motion is no more than 5 mm is selected. A CT series representing the average of the phases in the gating window is utilized for target delineation and treatment planning. Breath-hold CT in exhalation with IV (intravenous) and oral contrast is also obtained to assist with target and organ at risk delineation.

Gross tumor volumes (GTV) are generated on simulation imaging and encompass the primary tumor and any regional lymph nodes deemed to be involved based on size and/or metabolic activity on diagnostic PET. Internal target volumes (ITV) are generated to encompass residual motion visualized on 4-dimensional CT within the gating window. Clinical target volumes are created using a 5-mm margin, excluding adjacent GI structures. Planning target volumes (PTV) are generated using a 3-mm expansion in all directions, with additional margin (up to 5 mm) in the beam direction to account for range uncertainty. Treatment planning is performed using Syngo, which is a dedicated system for the IONTRIS, with an inverse optimization algorithm. The local effect model (LEM) [15, 16] is used to calculate biological equivalent doses (GyRBE). Various beam configurations are explored, with the general intent of combining posterior oblique and lateral beams. Inverse planning is performed using constraints described below.

CIRT is delivered using the IONTRIS system, manufactured by Siemens Medical Solution Health Service Corp. This system produces carbon treatment beams with a maximum field size of 20 cm × 20 cm in 3D modulated raster-scanning mode. Entrance energies range from 86 MeV/u to 430 MeV/u, and the scanning spot diameter ranges from approximately 3 mm to 14 mm. An internal ripple filter is used to broaden the Bragg Peak to 3 mm.

Each treatment room has a robotic couch for patient positioning with 6 degrees of freedom, an x-ray imaging system for 3D patient alignment, a laser system for precision patient positioning, and an interface for respiratory gating using the Enzai system, which employs an abdominal bell with a pressure sensor.

### Evaluating Dosimetric constraints

Dosimetric constraints for gastrointestinal organs treated with a 12-fraction CIRT schedule were based on a recent report from NIRS examining predictors of ulcer formation [17]. Biologically equivalent constraints for 14 and 17-fraction schedules were calculated using the Linear Quadratic Model, with α/β = 3 Gy. These constraints are summarized in Table 1. As viscous gastrointestinal organs are considered to function as serial structures with respect to radiation toxicity, we considered the 1 cc constraints to be “critical” constraints.

**Table 1:** Dose-volume constraints for GI organs (duodenum, stomach, and small bowel) in 12-, 14-, and 17-fraction regimens. Constraints in bold (first row) were considered to be “critical”. V_###GY_ represents the volume of GI organs receiving dose of ###Gy and higher

12 Fractions	14 Fractions	17 Fractions	Volume Limit
**V**_**50Gy**_	**V**_**53.1Gy**_	**V**_**56.6Gy**_	**< 1 cc**
**V**_46Gy_	**V**_48.6Gy_	**V**_51.9Gy_	< 2 cc
**V**_30Gy_	**V**_31.5Gy_	**V**_33.3Gy_	< 6 cc
**V**_20Gy_	**V**_20.8Gy_	**V**_21.8Gy_	< 24 cc
**V**_10Gy_	**V**_10.2Gy_	**V**_10.6Gy_	< 102 cc

We identified 13 consecutive patients who were treated with CIRT for LAPC at our institution. For each patient, we created three plans, with the following prescription doses:

- A - 4.6 GyRBE × 12 fractions = 55.2 GyRBE
- B - 4.0 GyRBE × 14 fractions = 56.0 GyRBE
- C - 3.0 GyRBE × 17 fractions = 51.0 GyRBE

Schedule A has been utilized in recent Japanese experiences [14, 17]. The fraction size of 4.0 GyRBE in Schedule B was considered in the initial design of our prospective trial comparing CIRT and photon radiotherapy in this setting. Schedule C was finally selected for our trial based on initial planning exercises.

The two most commonly used relative biological effectiveness (RBE) calculation methods for CIRT are the LEM model (used in this study) and the methods (first Kanai and later MKM) [18, 19] used at NIRS. Differences between the two systems have been described previously [16]. In the NIRS approaches (both Kanai and MKM), RBE was first derived based on the dose required to kill 90% of human salivary gland cells and then adjusted using RBE data obtained from their neutron therapy experience. RBEs for different fraction sizes were then linearly rescaled. In the LEM model, RBEs are calculated for any fraction size and not restricted to a specific survival fraction. The two models yield similar predictions with doses of approximately 4.5 GyRBE. Differences at other doses will lead to different delivered physical dose by the two systems with the same prescribed and planned biological equivalent dose outside the region where the same RBE is predicted by both systems. In general, if an LEM user wishes to apply an NIRS dose schedule of NIRS, corrections should be introduced (note that a single factor is insufficient to precisely convert a biological equivalent dose distribution). In this dosimetric study, we focused only on the applicability of a reported clinically effective dose schedule (in GyRBE), and the effects of differences in RBE modeling are outside the scope of this study.

For each patient and dose schedule, we generated a treatment plan that provided adequate target volume coverage (95% of the CTV receiving 95% of the prescription dose and 90% of the PTV receiving 90% of the prescription dose) and aimed to satisfy the normal tissue constraints listed in Table 1 for the duodenum, stomach, and small bowel. We assessed the ability to achieve each constraint (*n* = 5) for each patient (*n* = 13), organ (*n* = 3), and dose schedule (*n* = 3). Rates of achieving dosimetric goals were presented as percentages, with 95% confidence intervals calculated using a binomial distribution. For dose schedules where critical organ at risk constraints were not always met, we utilized logistic regression models to evaluate the probability of achieving those constraints as a function of distance from the GTV. We used a bootstrap resampling method (5000 iterations) to formulate 95% confidence bounds for the probability of achieving dosimetric constraints and minimize the influence of outliers in the data.

This work was approved by the Shanghai Proton and Heavy Ion Center Institutional Review Board. (Approval #: 171023EXP-01).

## Results

**Fig. 1:**
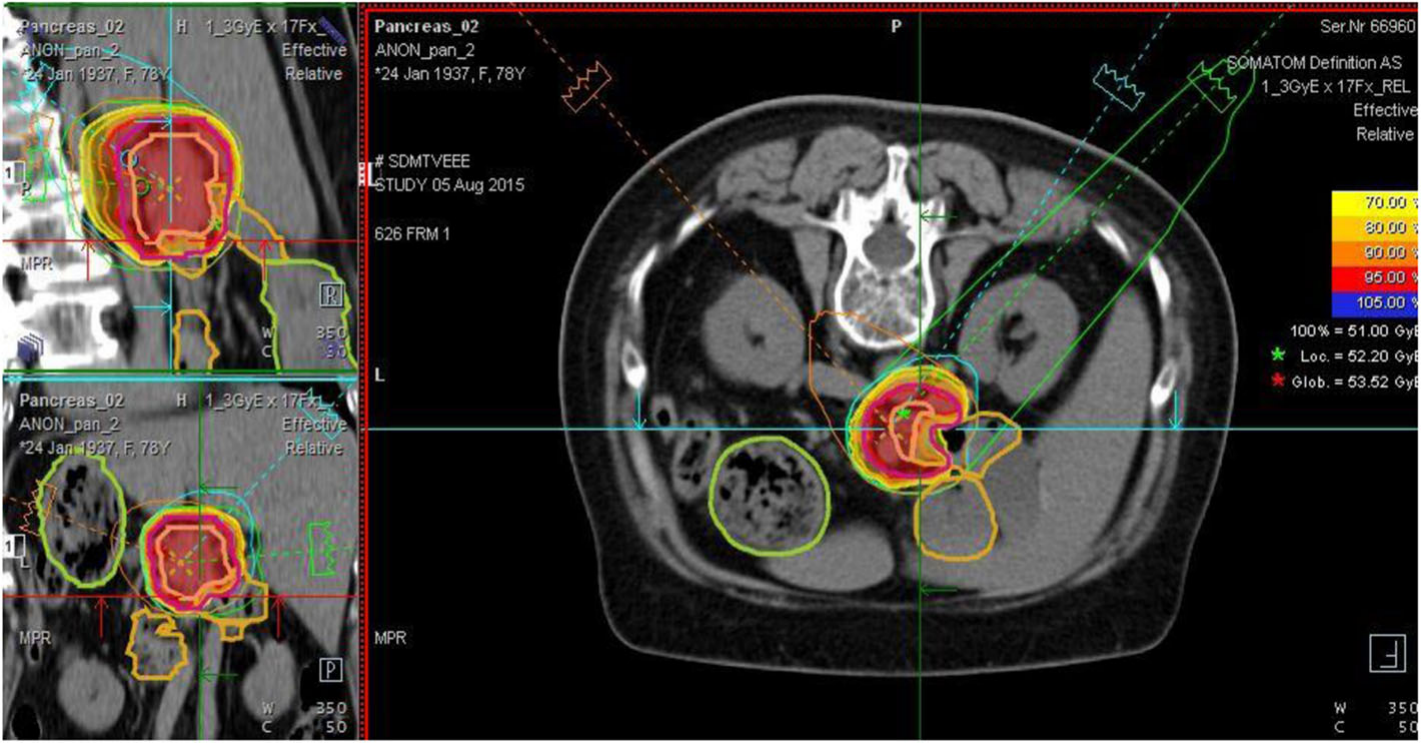
CIRT plan (3.0 GyRBE × 17) for LAPC

**Fig. 2:**
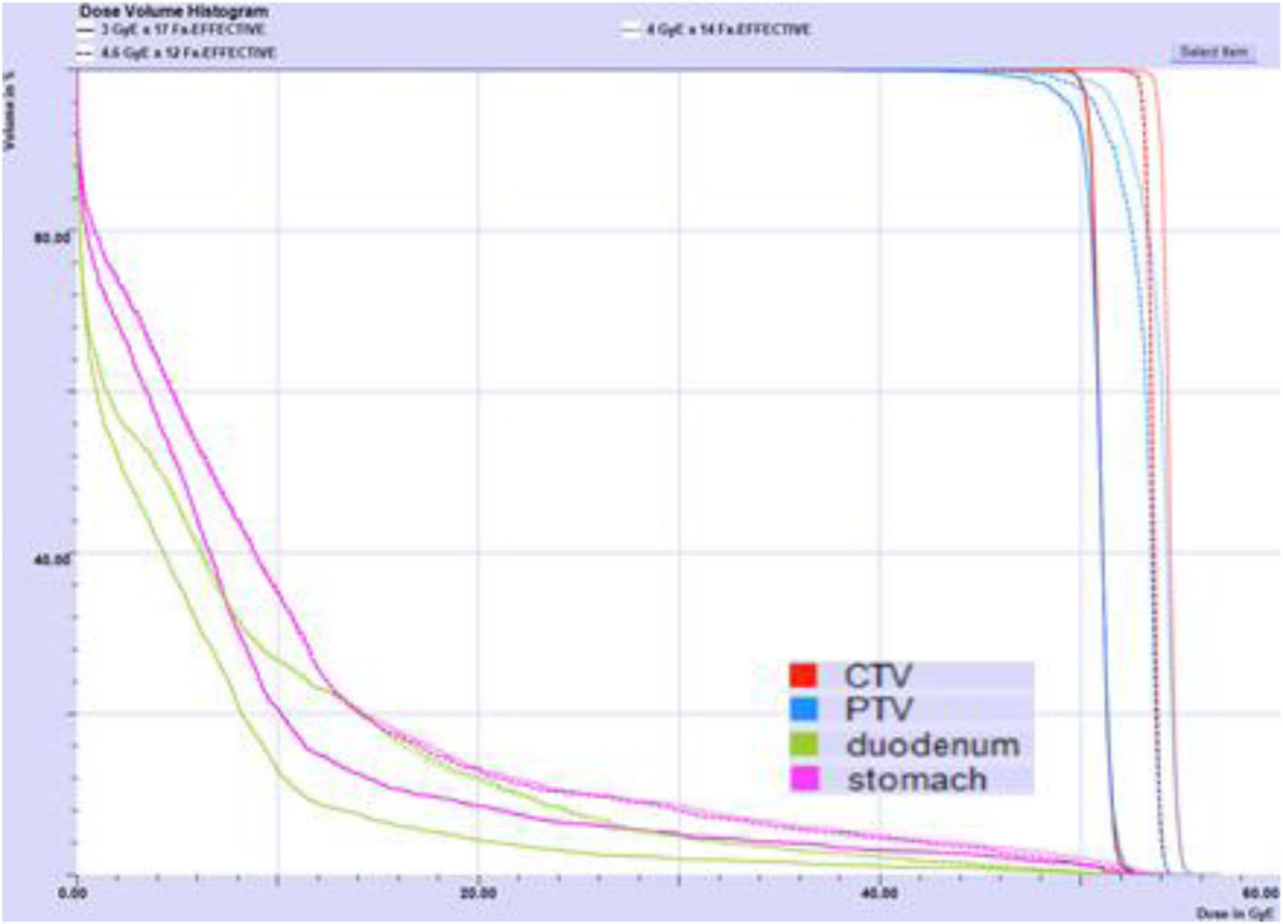
Dose-volume Histogram of target and critical structures

Patient and tumor characteristics are summarized in Table 2. Approximately one half of patients’ tumors were in the pancreatic head, and tumor diameters ranged from 3.3 cm to 9.5 cm. Figure 1 depicts a typical CIRT plan, generated using three posterior-oblique fields. The corresponding DVH is presented in Fig. 2.

**Table 2:** Patient Characteristics

Characteristics	Value
Total number of patients	13
Sex	
Male	7 (54%)
Female	6 (46%)
Age - range (median)	38-78 (66)
AJCC/UICC Stage	
IIA	2 (15%)
IIB	1 (8%)
III	10 (77%)
Tumor location	
Head	6 (46%)
Neck	1 (8%)
Body	3 (23%)
Body & Tail	3 (23%)
Tumor Diameter (cm) - range (median)	3.3-9.5 (6.1)
Tumor Size (cm^3^) - range (median)	14.8-134.9 (61.9)
CA-199 before RT (U/ml) - range (median)	14.3->1000 (68.4)

Tumor locations within the pancreas as well as distances from GTVs and CTVs to gastrointestinal organs at risk are detailed in Table 3. GTVs were immediately adjacent to the duodenum, stomach, and small bowel in 9, 8, and 5 out of 13 cases, respectively. CTVs were immediately adjacent to or overlapping with the duodenum, stomach, and small bowel in 12, 12, and 8 out of 13 cases, respectively.

**Table 3:** Tumor locations and minimal distances from GTVs (top) and CTVs (bottom) to gastrointestinal organs at risk (in mm)

	**PT1**	**PT2**	**PT3**	**PT4**	**PT5**	**PT6**	**PT7**	**PT8**	**PT9**	**PT10**	**PT11**	**PT12**	**PT13**
Duodenum	0	0	0	4.3	0	0	0	2	3.2	0	0	1.9	0
Stomach	0	23.3	0	0	12.4	2	6.1	0.9	0	0	0	0	0
Small Bowel	0	58	2	32.2	14.6	0	0	10.8	4.8	0	0.7	0	53.9
Tumor location	Body/tail	Head	Head	Head	Head	Head	Head	Body/tail	Head	Body/tail	Head	Body	Body
	**PT1**	**PT2**	**PT3**	**PT4**	**PT5**	**PT6**	**PT7**	**PT8**	**PT9**	**PT10**	**PT11**	**PT12**	**PT13**
Duodenum	0	0	0	2	0	0	0	0	0	0	0	0	0
Stomach	0	16.6	0	0	0	0	0	0	0	0	0	0	0
Small Bowel	0	52	0	27.5	10.3	0	0	4.4	0	0	0	0	47.8
Tumor location	Body/tail	Head	Head	Head	Head	Head	Head	Body/tail	Head	Body/tail	Head	Body	Body

**Fig. 3:**
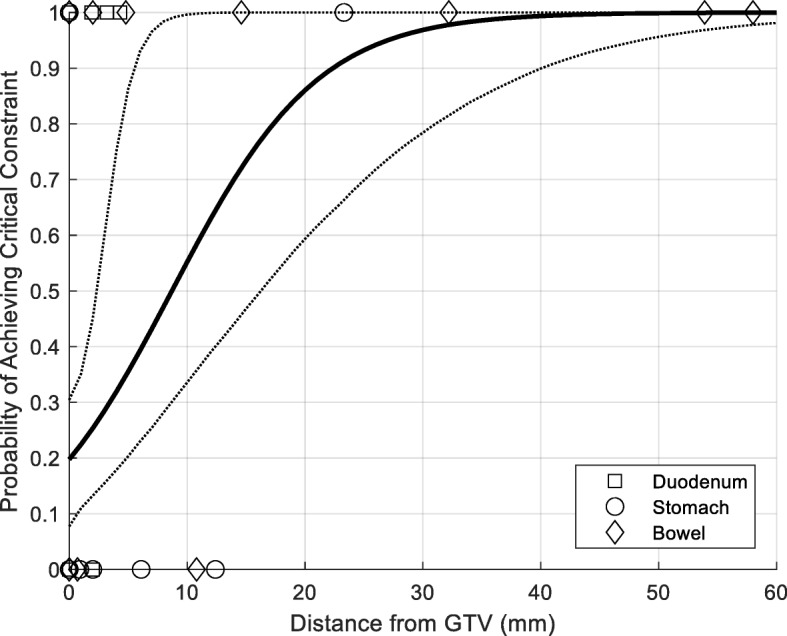
Logistic modeling describing the probability of achieving critical dosimetric constraints as a function of distance between the GTV and the organ at risk for Schedule A. The solid line depicts median probability based on 5000 bootstrap iterations; dotted lines represent 95% confidence intervals

**Fig. 4:**
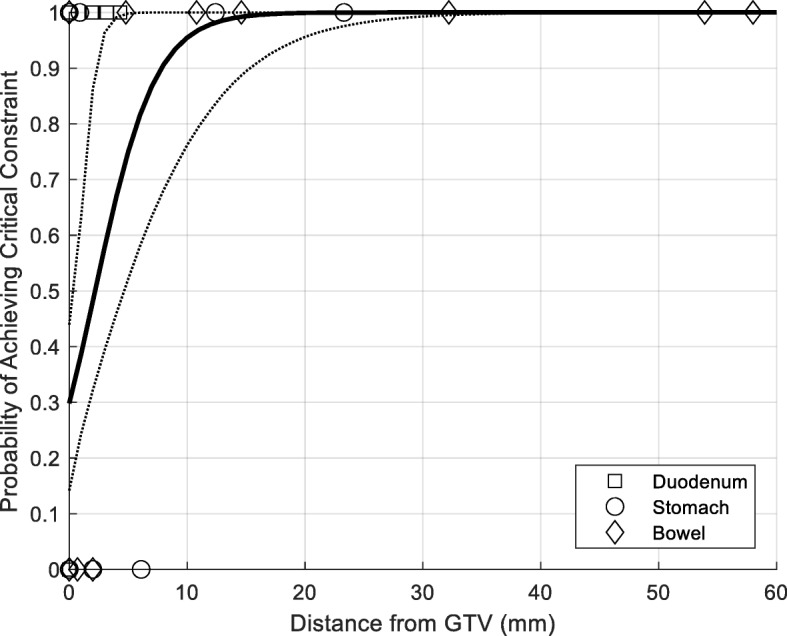
Logistic modeling describing the probability of achieving critical dosimetric constraints as a function of distance between the GTV and the organ at risk for Schedule B. The solid line depicts median probability based on 5000 bootstrap iterations; dotted lines represent 95% confidence intervals

Treatment planning results are summarized in Table 4. Entries that exceed tolerance are shaded in the tables. For each dose schedule, 195 constraints were evaluated (13 patients × 3 organs at risk × 5 constraints). Using dose schedules A, B, and C, 46% (95% CI: 39 to 53%), 49% (95% CI: 42 to 56%), and 77% (95% CI: 70 to 83%) of constraints were achieved, respectively. Rates of meeting “critical” constraints were 33% (95% CI: 19 to 50%), 49% (95% CI: 32 to 65%), and 100% (95% CI: 91 to 100%) for schedules A, B, and C. Of note, there was no patient for whom all of 15 organs at risk constraints could be achieved with any of the dose schedules we explored. Logistic modeling results for Schedules A and B are depicted in Figs. 3 and 4. For Schedule A, 22 mm of separation (95% CI: 6 to 40 mm) between the GTV and an organ at risk is required to provide a 90% chance of treating that lesion “safely”. For Schedule B, this separation was reduced to 8 mm (95% CI: 2 to 15 mm). Modeling was not performed for Schedule C, as critical constraints were met in all cases using that dose schedule.

**Table 4:**
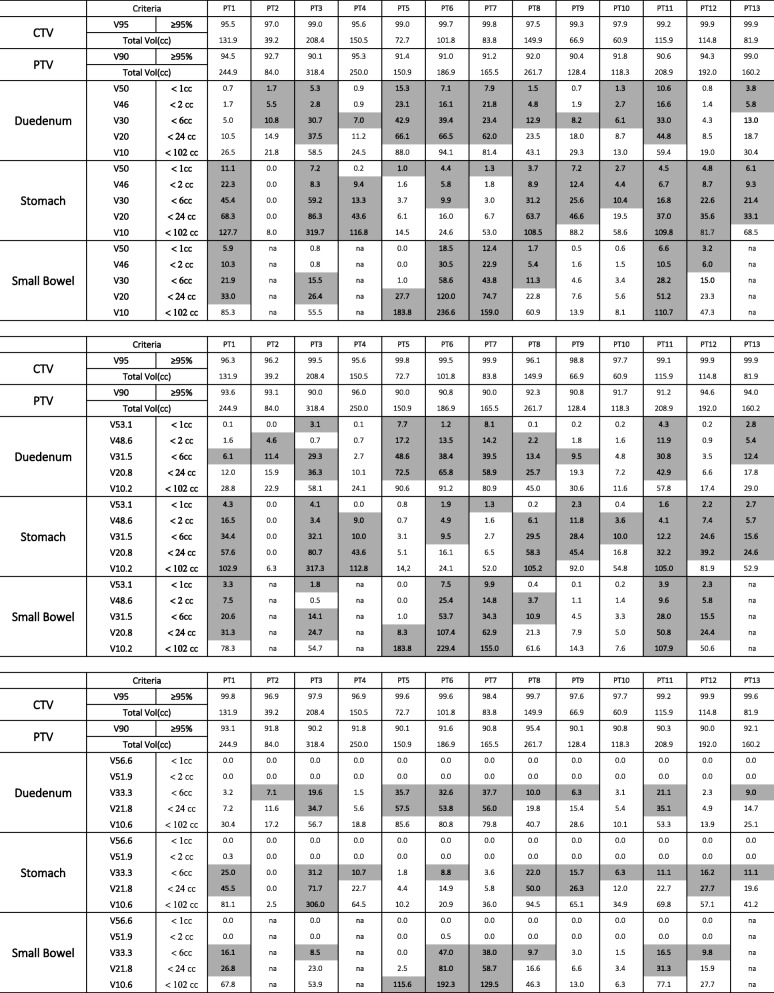
Treatment planning results for dosing schedules of 4.6 GyRBE x 12 (top), 4.0 GyRBE x 14 (middle), and 3.0 GyRBE x 17 (bottom). Shaded regions indicate constraints that were not met

## Discussion

We performed detailed dosimetric analyses using a series of unselected patients who were treated with CIRT for LAPC. We found that critical (i.e., high dose) dosimetric constraints for the duodenum, stomach, and small bowel are often not achievable with fraction sizes of 4.6 GyRBE or 4.0 GyRBE but can be met using daily fractions of 3.0 GyRBE. As expected, the likelihood of meeting dosimetric constraints is also related to the distance between the tumor and organs at risk. Reducing the daily fraction size is predicted to improve our ability to safely deliver CIRT for broad populations of patients with pancreatic cancer.

While it may not be surprising that reduced daily radiotherapy doses and increasing distance between the target lesions and surrounding organs at risk would reduce the predicted risk of normal tissue injury, we believe that this analysis highlights critical considerations in the clinical implementation of novel forms of radiotherapy. Should promising yet potentially toxic forms of treatment be tested exclusively in subjects with favorable anatomic/biologic parameters? Should broader patient populations be enrolled and treated as completely as possible (e.g., with a “dose-painting” approach)? Should phase I studies in Radiation Oncology focus on incrementally increasing the normal tissue dose rather than the tumor dose [20]? In non-small cell lung cancer, a radiotherapy dose that was deemed to be safe and effective in early phase studies (74 Gy) was found to *shorten* overall survival compared to a standard dose of 60 Gy in a large randomized trial [21]. That experience highlights the need for randomized trials to establish new treatment techniques.

Our findings may seem to contradict recent reports from Japan, where a CIRT regimen of 4.6 GyRBE × 12 (Schedule A, in the present analysis) was delivered with concurrent gemcitabine and deemed to have an acceptable safety profile [14]. We believe that this can be reconciled by comparing the patient populations in the two studies. Patients were excluded from the Japanese trial if they had “direct invasion of a tumor into the mucosal surface of the gastrointestinal tract or had received a metal stent insertion as treatment for obstructive jaundice.” The median tumor volume in the Japanese series was 14.8 cm^3^, which was the smallest tumor size in our series. Additionally, the largest tumor volume in the Japanese series was approximately equal to the median tumor volume in our series. While it was reasonable for NIRS investigators to select patients carefully as they explored a novel and potent form of radiotherapy, we now hope to extend CIRT to a broader population of patients with LAPC. Our analysis suggests that this may be achieved safely with a modest reduction of the daily fraction size.

In a second report, the Japanese group explored dosimetric predictors of gastrointestinal toxicity [17]. All plans generated in that series met their ‘critical’ constraint, which was to treat less than 2 cm^3^ of gastrointestinal organs with doses exceeding 46 GyRBE. Twelve out of 58 patients evaluated developed ulcers, mostly grade 1–2. Ulcer formation was found to be related to low/medium dose spillage into organs at risk, and new constraints for limiting this spillage were recommended. We found that these constraints could rarely be met in our patient cohort, which again is likely a result of patient selection. Further work to elucidate the importance of these constraints is warranted, especially since the toxicities observed in the Japanese series were mostly low-grade.

Reducing the fractional dose with protracted fractionation is a common strategy in radiation therapy to reduce normal tissue toxicities if the difference in radiation sensitivity reflected by the α/β ratio between a tumor and normal tissues, is sufficiently large, which is the case in here, as the α/β has the value of 3 Gy for GI late toxicity and 6.7 Gy for pancreatic cancer [22]. The use of photon-based α/β values is justified as in theory all doses in CIRT are expressed in photo-equivalent Gy through RBE modeling. The ultimate accuracy and efficacy can only be determined by clinical studies.

Determining the RBE for CIRT poses numerous challenges. RBE calculation methods are different between the NIRS’s planning system and Syngo (the planning system used in this study). It has been shown that with a fraction size of approximately 4.6 GyRBE, both systems agree reasonably well [22, 23]. At other fraction sizes, dose-dependent correction factors may be required to convert doses across models. Additional variation in RBE might result from beam delivery types, namely broad-beam with range-modulator or scanning beam, implying that clinical data obtained from one type of beam delivery system might not be totally compatible when using other beam delivery type. No matter how they are calculated, RBE values are based on laboratory experiments and must be validated as predictors of clinical outcomes. It should be reiterated that comparisons in this study are based only on doses in GyRBE, with one reference dose schedule of 4.6 GyRBE × 12 that was reported with clinical data using broad-beam. Our plans were developed based on GyRBE optimization. Differences between our RBE calculation method and algorithms employed at other institutions should not negate the key conclusion of this dosimetric study – that the safety of CIRT is a function of both treatment schedule and patient anatomy. As is true for many modeling and dosimetric studies, our findings simply provide guidance for treatment strategies that must be tested in clinical trials.

We conducted a Phase I study to establish the safety of CIRT using a daily fraction size of 3.0 GyRBE in LAPC (results in preparation for publication). Our trial follows a “3 + 3” design and employs a combination of photon radiotherapy and CIRT in the first two dose levels. The initial dose escalation plan is described in Table 5. Another study design that may be appropriate for early-phase CIRT trials is the TITE-CRM model, which may be better suited to account for subacute and delayed adverse events [24, 25].

**Table 5:** Dose levels in an ongoing clinical trial testing the safety and feasibility of carbon ion radiotherapy with concurrent chemotherapy for locally advanced pancreatic cancer

Dose Level	Photon Dose	Carbon Ion Dose	Total dose	BED6.7
1	1.8 Gy × 9	3.0 GyRBE × 10	46.2 GyRBE	64.0 GyRBE
2	1.8 Gy × 5	3.0 GyRBE × 12	45.0 GyRBE	63.5 GyRBE
3	–	3.0 GyRBE × 15	45.0 GyRBE	65.1 GyRBE
4	–	3.0 GyRBE × 16	48.0 GyRBE	69.5 GyRBE
5	–	3.0 GyRBE × 17	51.0 GyRBE	73.8 GyRBE
6	–	3.0 GyRBE × 18	54.0 GyRBE	78.2 GyRBE
7	–	3.0 GyRBE × 19	57.0 GyRBE	82.5 GyRBE
8	–	3.0 GyRBE × 20	60.0 GyRBE	86.9 GyRBE

To conclude, while selected patients with LAPC may be treated safely with a CIRT regimen of 4.6 GyRBE × 12, our dosimetric analyses indicate that for a given technical setting (type of beam delivery system, RBE model, and target volume specification) a more conservative schedule may be required to safely treat a broader population of LAPC patients. This concept was used to guide our first Phase-I clinical trial.

## Data Availability

The data that support the findings of this study are available in the institution’s clinical database but restrictions apply to the availability of these data, which were used under license, and so are not publicly available. Data are however available from the authors upon reasonable request.

## Abbreviations

CIRT: Carbon ion radiotherapy
GTV: Gross tumor volumes
ITV: Internal target volumes
LAPC: Locally advanced pancreatic cancer
LME: Local effect model
NIRS: National Institute of Radiological Sciences
PTV: Planning target volumes
RBE: Relative biological effectiveness
